# The Treatment of Pneumonia in Children

**Published:** 1894-10

**Authors:** Frank S. Parsons

**Affiliations:** Philadelphia, Pa.; Formerly Lecturer on Children’s Diseases in the College of Physicians and Surgeons, Boston


### THE TREATMENT OF PNEUMONIA IN CHILDREN.

BY FRANK S. PARSONS, M.D., PHILADELPHIA, PA.

Formerly Lecturer on Children's Diseases in the College of Physicians and Surgeons, Boston.

It will be necessary for a clear understanding of pneumonia in children to consider the two great varieties under separate heads: viz. croupous pneumonia and catarrhal or broncho-pneumonia.

So great are the differences in the etiological and therapeutic factors in these two forms, that they should be considered as distinct diseases.

Croupous pneumonia is, at present, generally considered an infectious disease, determined by a specific cause, and for this reason deserves to be classed among the disease termed "zymotic." It will, if untreated, run a definite course in a regularity of time, and its natural tendency is to recovery. The self-limitation of this form of pneumonia would imply the production in the blood of an agent which is antagonistic to the specific cause of the disease—an anti-pneumotoxin.

If, as German scientists have demonstrated, after croupous pneumonia has progressed a few days there appears in the blood this agent antagonistic to the pneumococcus, our future dissertations on this subject will be exceedingly short. The sick will be made to heal the sick, and the physician, armed with hypodermic needle, will act as mediator between them. But these beautiful theories, unfortunately, like many others over which the world has gone wild, can only be made theoretical, and seem to contain no practical value.

To take up your valuable time enumerating the various therapeutic measures employed in the treatment of this disease, is not my intention in this paper, and I shall only consider those which have seemed to me most advantageous, recognizing that there are other therapeutic roads which lead to the same result.

Given, then, a disease which we believe to be specific, self-limited and dangerous to a degree comparative with the intensity of its symptomatic indications, and the capability of the heart to bear up under the greater strain laid upon it, what are our therapeutic indications in treating such a malady ?

As before mentioned, the antidotal remedy, except as it naturally is produced in the blood, has not been found practical in application as yet, and it is doubtful if, by outward applications, or remedies usually employed internally, we are ever able to abort a case of true croupous pneumonia. Congestions of the lungs, which may give rise to consolidated areas on percussion, are quite another matter.

In croupous pneumonia of children the relative size of the bronchical tubes being larger, and the alveoli smaller, than in the adult, congestions of the interstitial structures occasion more profound results. The fever is apt to be higher, the nervous symptoms greater, and oxygenation lesser.

First, then, we should endeavor to promote diaphoresis, and an increased elimination of carbonic acid gas with a reduction of temperature.

Second, we should promote elimination through the kidneys by diuresis.

Third, we should promote an increased alkalinity of the blood, thereby lessening the fibrin and tendency to coagulation.

Fourth, we should quiet the nervous system and favor sleep.

A large, well ventilated room, not too much darkened, with an average temperature of about 70° F., is a necessary, but, unfortunately, not always convenient, sanitary measure which will prove of advantage.

As a rule very little medicine is needed in the treatment of croupous pneumonia of children. When the temperature is high, skin hot and dry, and there is a good deal of restlessness, two or three drops of the compound tincture of ipecac ("the liquid Dover's powder") every hour or two until there is marked improvement, will be of benefit. To promote diaphoresis and diuresis there is nothing better than the old stand-by, sweet spirits of nitre (spiritus etheris nitrosi). Acetate of potash may be given to promote alkalinity in the blood and guard against clot-formation. Liquor ammonii acetatis will greatly assist the elimination through the skin and cause a reduction of temperature.

The following formula given by Dr. Larrabee, of Louisville, Ky., has proved a great benefit in croupous pneumonia in my experience, viz.:

℞ Spts. etheris nitrosi,
Potassæ acetatis aa . . . . . . . . . . . . . . . . . . . . ʒ iss.
Spts. mendereri,
Aquæ camphoræ, aa . . . . . . . . . . . . . . . . . . . . ℥ iii.
To be left with slightly acid reaction to litmus.
M. Sig.—Teaspoonful every two hours to a child.

Routine treatment in this disease should be avoided. Individual cases must be watched closely and often.

*Temperature.*—When the temperature is high, in my opinion the warm bath is to be preferred to the cold. It certainly is more grateful to the little sufferer, and the shock of a cool plunge is obviated. After the warm bath the chest may be enveloped in a warm wet pack, when the child will be almost certain to break out in a gentle perspiration, which is followed by refreshing sleep.

The coal-tar products, as antipyretics, have, of late, fallen into more or less disrepute in the treatment of pneumonia; the difficulty seems to lie in the supposition that they deoxidize the blood.

Between aconite and veratrum in the early stages, the majority of practitioners prefer veratrum, which is considered safer. I believe there is little preference practically, but my experience has led me to rely more on aconite of the two. Care should always be exercised in the employment of either.

*Expectorants.*—In croupous pneumonia no expectorant is needed. The fluidity of the mucous collections can as easily be kept up with cold water for a refreshing beverage as to nauseate the patient with composite mixtures of ipecac, squills, senega, ammonia, and opium.

*Purgatives.*—It is a common observation that purgation in the stage of hepatization is bad. Probably this results from the general depression resultant upon the administration of the drug.

*Stimulation.*—Death in croupous pneumonia is nearly always from a failure of the cardiac muscles; the right side of the heart is engorged with blood in its endeavor to force the vital fluid through the obstruction present in the lungs. This causes the right ventricle to become distended so that the apex beat may be displaced. Clearly, then, our plan of stimulation of the heart lies not so much with the necessity for goading the organ to more work, but that we make the extra work it has to do lighter. We should relieve the engorgement by dilatation of the capillaries. Our forefathers recognized this when they averred that "bleeding is good in pneumonia." While we do not need to revive this old-time custom at the present day, because we can accomplish the same thing with less danger to our patients, yet there is an underlying principle about this that we will do well not to lose sight of, viz., the relief of an overburdened heart. Our object in this line may be attained by direct application of heat to the chest by means of the warm pack. We will find that drugs of the belladonna class will diminish the blood pressure by dilating the capillaries. Nitroglycerine will often rescue a heart that is overcharged with venous blood in the same manner as aspiration. Digitalis should not be used before the crisis. Alcohol has great value as a cardiac stimulant in this disease, by reason of its power to dilate the capillaries and its action on respiratory centers. Strychnine in doses of 1-300 of a grain every four hours is an excellent heart tonic in pneumonia of children.

*Applications to the Chest.*—Beyond the soothing effect of heat and its stimulating effect on the heart, there is little benefit to be derived from the application of poultices. Hot water may be applied by means of cloths wrung out in the same and confined about the chest with cotton or oiled silk. Probably they have no influence on the lesion, except as an agent to determine the blood to the skin, and thus relieve the heart.

*Nervous Symptoms.*—For the pain, irritating cough, and sleeplessness, the drugs classed as antipyretics are occasionally useful. Phenacetine holds, perhaps, the first place among this class of drugs. From a half to two grains of the drug can be given infants, according to age, but should be used only for the nervous phenomenon, irrespective of temperature.

Catarrhal pneumonia, or, better termed, broncho-pneumonia, is an entirely different disease. While there may be interstitial consolidation with the coexisting bronchitis, its etiology, symptomatology, and treatment are entirely different from the croupous variety.

This form is far more common in the young infant than the specific; a fact which is probably due to the ease with which a catarrhal inflammation extends along the respiratory tract, and the comparative immunity that very young infants enjoy from infectious diseases.

The child sick with broncho-pneumonia should be clad in a long, warm nightdress made of cotton flannel and kept in the same, unless necessary to change for the sake of cleanliness, until the severity of the disease has passed. A chest protector of eiderdown flannel should be made and worn. I prefer this kind of protector on account of the thickness, softness, and warmth which it affords, and believe it is of more value than all the poultices or sinapisms that are usually applied.

For the fever in this form of pneumonia chloral hydrate is coming into good repute. As an antipyretic this drug possesses admirable qualities. It is an antiseptic, volatile, and has the slightest solubility in the blood, and hence eliminative. If it is not pushed too far it is not toxic.

In this form of pneumonia it is necessary to treat the cough. This should require a careful consideration of the condition of the patient. In broncho-pneumonia we have a stasis of the expulsive powers of the bronchi due to thickness and viscidity of the mucus and the weakened condition of the muscles of the tubes from exhaustive attempts to cough up the secretion during the pre-existing bronchitis. Therefore we must first stimulate the mucous glands to increase the watery elements of the secretion. For this the steam spray is an excellent adjuvant, but the most reliable drug for this purpose is ipecac. Combined with the latter to relieve the irritability and restlessness some form of opium is advantageous, and that we may get free elimination and a good action of the kidneys, I generally add citrate of potash.

For the foregoing reasons I have been in the habit of prescribing the following formula:

| | | |
|---|---|---|
| ℞ | Vini ipecac . . . . . . . . . . . . . . . . . . . . . . . . | ʒ i |
| | Potass. citratis . . . . . . . . . . . . . . . . . . . . . . . . | gr. 30 |
| | Tinct. opii camph. . . . . . . . . . . . . . . . . . . . . . . | ʒ ii |
| | Elixir simplicis . . . . . . . . . . . . . . . . . . . . . . . . | ℥ i |
| | Aqu ædistil. q. s. ad. . . . . . . . . . . . . . . . . . . . . . | ℥ iv |

M. Sig.—A teaspoonful to an infant six months old every two hours.

Stimulating expectorants, so-called, are sometimes useful in the latter stages of this disease, but are useless in the earlier stages.

Much benefit will be obtained by the boiling in the sick room of a kettle of water; the steam arising therefrom tends to ease the cough by facilitating expectoration. Turpentine may be added if desired.

The bronchial tubes, stomach and intestinal canal will be relieved of the mucus and muco-pus by an occasional emetic or a little calomel, given cautiously, but not in sufficient quantity to tax the strength of the child. I have seen good results in clearing the intestinal tract by allowing the child to drink freely of water into which has been put a little of Marchand's "Hydrozone" in the proportion of a teaspoonful to the half pint of water. If used too strong the hydrozone will occasion decomposition of the pus into painful quantities of carbonic acid gas, causing colic, but if this destruction of pus is carried on less vigorously by a larger dilution, the pain or discomfort is obviated. The action of the hydrozone is one simply of cleansing the mucus membrane.

*Stimulation.*—The heart tonics necessary in this form of pneumonia do not differ from those indicated in the croupus variety, except that tde objections to digitalis are removed in broncho-pneumonia; in fact, together with whisky or brandy, we are quite in need of it. It is always a good plan to combine any depressing antipyretic with a drop or two of digitalis in bronchial inflammation in children. It is spur and oats to a flagging heart where not contraindicated by any extensive consolidation of lung tissue.

Malarial element may be occasionally found in these bronchial affections in children, and if suspected, should receive proper attention.

In broncho-pneumonia, the result of intestinal disturbance and the bacterium coli communis, Sevestre employs calomel in one-grain doses to children under six months. To relieve the lung, dry cupping or mustard plasters are used; for dyspnea two or three injections of ether or of caffeine each day and alcohol rubbings for the algid tendencies are advised.

The complications of pneumonia, pleurisy, and cerebral symptoms are to be met by revulsive treatment, and the bromides and chloral with ice to the head.

The diet in this disease should be fluid, chiefly of milk.

